# The Protective Effects of γ-Tocotrienol on Muscle Stem Cells Through Inhibiting Reactive Oxidative Stress Production

**DOI:** 10.3389/fcell.2022.820520

**Published:** 2022-03-15

**Authors:** Shuo Yang, Juan Yang, Huiwen Zhao, Rong Deng, Hancheng Fan, Jinfu Zhang, Zihao Yang, Huihong Zeng, Bohai Kuang, Lijian Shao

**Affiliations:** ^1^ Department of Occupational Health and Toxicology, School of Public Health, Nanchang University, Nanchang, China; ^2^ Jiangxi Provincial Key Laboratory of Preventive Medicine, School of Public Health, Nanchang University, Nanchang, China; ^3^ Department of Biological Genetics, School of Basic Medicine, Nanchang University, Nanchang, China; ^4^ Department of Histology and Embryology, School of Basic Medicine, Nanchang University, Nanchang, China; ^5^ Jiangxi Provincial Key Laboratory of Interdisciplinary Science, Nanchang University, Nanchang, China

**Keywords:** Duchenne muscular dystrophy, γ-tocotrienol, muscle stem cells, oxidative stress, cellular proliferation

## Abstract

Pseudotrophic muscular dystrophy is a common clinical skeletal muscle necrotic disease, among which Duchenne muscular dystrophy (DMD) is the predominant. For such diseases, there is no clinically effective treatment, which is only symptomatic or palliative treatment. Oxidative stress and chronic inflammation are common pathological features of DMD. In recent years, it has been found that the pathophysiological changes of skeletal muscle in DMD mice are related to muscle stem cell failure. In the present study, we established a DMD mice model and provided tocotrienol (γ-tocotrienol, GT3), an antioxidant compound, to explore the relationship between the physiological state of muscle stem cells and oxidative stress. The results showed that the application of GT3 can reduce ROS production and cellular proliferation in the muscle stem cells of DMD mice, which is beneficial to promote the recovery of muscle stem cell function in DMD mice. GT3 treatment improved the differentiation ability of muscle stem cells in DMD mice with increasing numbers of MyoD^+^ cells. GT3 application significantly decreased percentages of CD45^+^ cells and PDGFRα^+^ fibro-adipogenic progenitors in the tibialis anterior of DMD mice, indicating that the increased inflammation and fibro-adipogenic progenitors were attenuated in GT3-treated DMD mice. These data suggest that increased ROS production causes dysfunctional muscle stem cell in DMD mice, which might provide a new avenue to treat DMD patients in the clinic.

## Introduction

DMD is a recessive neuromuscular disease linked to the X chromosome. The birth rate of affected men is about 1/3,600–6,000. The disease is mainly caused by frameshift deletion, meaningless or repeated mutations of the gene encoding dystrophin (dystrophin) on the X chromosome (Xp21.2) (Frank et al., 2020; Miyatake et al., 2016). Dystrophin is an important part of a protein complex, which connects the muscle cytoskeleton and extracellular matrix to maintain the integrity and stability of the muscle membrane (McGreevy et al., 2015). Dystrophin binds to F-actin through its cytoplasmic N-terminal domain. The extracellular C-terminal domain of dystrophin binds to β-dystrophic glycan, which acts as a bridge and anchor protein. The mutation of dystrophin leads to the disruption of dystrophin-related glycoprotein complexes, which causes membrane instability and sensitizes to various stresses and muscle fiber necrosis. DMD is a progressive disease (Ogura et al., 2014). Its pathological characteristics are progressive muscle damage and weight loss. The patient developed symptoms of muscle weakness when they are young. The motor function will gradually decline when they are growing up. The patients will gradually unable to walk independently and die of heart or respiratory failure around the age of 20 (Nakamura, 2019). The existing treatment approaches of DMD, such as steroid therapy, stem cell transplantation and gene therapy, have certain defects and limitations (Chamberlain and Chamberlain, 2017; Kole and Krieg, 2015; Lim et al., 2018).

Chronic inflammation and oxidative stress are common pathophysiological characteristics of DMD (Whitehead et al., 2010). Excessive ROS will negatively affect cell proliferation, resulting in cell death because of high levels of oxidative stress, damaging lipids, protein and DNA (Le Moal et al., 2017; Perry et al., 2000). Some research results indirectly support the existence of high levels of ROS in DMD muscle stem cells. The abnormal vascular distribution of DMD muscle leads to ischemia and hypoxia (Shimizu-Motohashi and Asakura, 2014). Therefore, increased ROS production may be one of the reasons for DMD muscle cell dysfunction. Recent studies have shown that pathophysiological changes of skeletal muscle in DMD mice models are related to muscle stem cells exhaustion. Muscle stem cells can be detected between the basement membrane and the muscle fiber membrane, which is in a quiescent status under steady state condition. They are the main source of regulating the growth and regeneration of new skeletal muscle and essential for the stability and regeneration of skeletal muscle throughout the life cycle (Bischoff, 1986). Cellular reactive oxygen species is a double product of oxidative phosphorylation, which can regulate physiological activities of stem cells (Scaramozza et al., 2019). On the other hand, there is evidence that the main effect of antioxidants on the muscles of DMD mice model is to inhibit cell death and promote muscle cell maturation by reducing oxidative stress (Sebori et al., 2018).

Vitamin E is a widely used natural supplement with strong antioxidant, neuroprotective and anti-inflammatory properties (Peh et al., 2017), which can regulate the peroxidation reaction and control the production of free radicals in the body. There are eight different subtypes of these compounds, which are collectively referred to as tocols (Singh et al., 2016; Zainuddin et al., 2017). It has always been believed that α-tocopherol subtype is the main subtype of vitamin E’s antioxidant and anti-inflammatory effects. There is conclusive evidence that γ-tocotrienol (GT3) is the advanced subtype of vitamin E, which has stronger antioxidant properties than the former (Palau et al., 2018; Yamashita et al., 2016). It was shown that stem cells can efficiently keep in an undifferentiated status after supplementation of antioxidants, which benefits to prevent stem cell exhaustion (Saheera et al., 2019). However, whether GT3 can enhance the function of muscle stem cells in DMD mice is still unknown. In this study, mdx mice were generated as a pathological model of DMD. GT3 treatment was used to evaluate whether GT3 can ameliorate the phenotypic and functional defects of muscle stem cells in the DMD mice model.

## Materials and Methods

### Animals

For this study, we used 40-week-old DMD knockout mice with a genetic background of C57BL/6J and ordinary C57BL/6J strain SPF male mice, weighing 26–28 g, from the Institute of Model Animals of Nanjing University. The animals were maintained on a regular 12-h day and night changes with free access to food and water during the whole experimental period. The environmental temperature was controlled at 23 ± 3°C and the relative humidity was maintained between 30 and 70%. The whole animal experiment process is strictly in accordance with the 3R principle of experimental animals to give the experimental animals humane care. Mice were divided randomly into four groups: wild type (WT), DMD knockout mice (DMD) and the two types of mice which were treated with GT3. The animal welfare and Ethics Committee of Nanchang University reviewed and approved the disposal method of the animals during the experiment (NCU-CLA-2019-319).

### γ-tocotrienol Treatment

γ-tocotrienol was purchased from Cayman Inc. (Item No: 10008494). γ-tocotrienol was maintained in the absolute ethanol with a concentration of 10 mg/ml. After drying with nitrogen, γ-tocotrienol powder was dissolved in the pre-prepared solvent to make the final concentration 4 mg/ml. The solvent is composed of anhydrous ethanol, Tween 80, polyethylene glycol 400, and ultrapure water with a ratio of 2:1:1:16. Mice were subcutaneously injected with GT3 (50 mg/kg). The same volume of solvent injection was used as controls. All mice were sacrificed at 48 h after treatment. Bilateral tibialis anterior were collected, weighed, and processed for further study.

### Histology and Immunostaining

Tibialis anterior were fixed in 4% PFA. Paraffin-embedded sections (5 µm) were collected. Hematoxylin and Eosin (HE) staining was performed according to a conventional staining procedure. The mean girth of muscle bundle in cross section was calculated.

The expression of Pax7 and proliferating cell nuclear antigen (PCNA) was assessed in muscle cells by immunohistochemistry staining. Citrate solution (pH = 6.0) is used for antigen retrieval and endogenous peroxidase was eliminated with 3% hydrogen peroxide. Sections were then washed three times in 1 × PBS, which was then followed by an overnight incubation at 4°C with mouse monoclonal primary antibody (Pax7, 1:100, Abcam ab55494; PCNA, 1:400, Boster Biological Technology co. ltd. BM0104; MyoD1,1:400, Affinity AF7733; PDGFRα, 1:200, Bioss bs-0231R). Slides were incubated with goat anti-mouse secondary antibody for 30 min at RT and followed by three washes in 1 × PBS. The color is developed by diaminobenzidine method. Pictures were taken under microscope (Olympus). Numbers of Pax7, PCNA MyoD1, PDGFRα positive cells were counted in the cross-section area.

### Quantitative Real-Time PCR

Total RNA was extracted from ipsilateral DMD and tibialis anterior of WT mice using Trizol reagent (Trans, Cat# ET111-01). A total of 1 μg of RNA was used for RT using the Reverse Transcription System (Cat#K1322; Thermoscientific) according to the manufacturer’s protocol. RT-PCR was conducted in triplicate determinants of PerfectStart Green qPCR SuperMix (Cat#AQ601; Trans) with the appropriate forward and reverse primers. For quantitative analysis of gene expression, results were averaged from three replicates in three independent experiments. Primers used for quantitative RT-PCR analysis are listed in Table 1. HPRT was used as an internal reference.

**TABLE 1:** Primer sequences for RT-PCR.

Primer sequences
Cyclin D1 F: 5′-ATG​TTC​GTG​GCC​TCT​AAG​ATG​AAG-3′
R: 5′-CAG​AAG​CAG​TTC​CAT​TTG​CAG​CAG-3′
Cyclin D2 F: 5′-CCA​GGA​GCT​GCT​GGA​GTG​GGA​ACT​G-3′
R: 5′-GCT​TGC​GGA​TCA​GGG​ACA​GCT​TCT​C-3′
P27 F: 5′-GGT​CTC​AGG​CAA​ACT​CTG​AGG​AC-3′
R: 5′-GCG​AAG​AAG​AAT​CTT​CTG​CAG-3′
HPRT F: 5′-AGC​AGT​ACA​GCC​CCA​AAA​TGG​TTA-3′
R: 5′-TCA​AGG​GCA​TAT​CCA​ACA​ACA​AAC-3′

### Measurement of Muscle Stem Cells by Flow Cytometry

The muscle tissue is grinded and digested in DMEM culture medium containing 10% FBS containing 1×collagenase/dispase enzyme (1 mg/ml; Roche) for 1 h at 37°C. The supernatant is filtered to lyse the red blood cells to obtain a single cell suspension. Cells were stained with PE anti-mice/rat CD29 Antibody, PerCP/Cyanine5.5 anti-mice CD184 (CXCR4) Antibody, APC anti-mouse CD45 Antibody, APC anti-mouse CD11b Antibody. DAPI was used to exclude dead cells. All flow antibodies were ordered form Biolegend Inc. Stained cells were analyzed by flow cytometry (BD FACSVerse). CD45^−^CD11b^−^CD29^+^CXCR4^+^ cells were used to define muscle stem cells. Percentages of CD45^−^CD11b^−^CD29^+^CXCR4^+^ cells in DAPI^−^ cells were demonstrated.

### ROS Production

Cells were stained with muscle stem cell antibodies. 20 μmol/L DCFDA (Invitrogen), a probe for intracellular ROS levels, was used to stain cells for 30 min at 37°C under dark conditions. Dead cells were eliminated by DAPI. Stained cells were analyzed using a flow cytometer (BD FACSVerse). Mean fluorescence intensity (MFI) of DCFDA in muscle stem cells was calculated using a FlowJo software.

### Statistics

All statistical analyses were performed using GraphPad Prism 8. Data were presented as mean ± SEM. Differences between groups are assessed using Student’s t-test and one-way or two-way analysis of variance (ANOVA), using Tukey’s multiple comparison test. Data were considered significant differences from the mean in control when *p* < 0.05.

## Results

### Loss of Dystrophin Results in Increased Myocyte Proliferation and Hypertrophy of Skeletal Muscle

**FIGURE 1:**
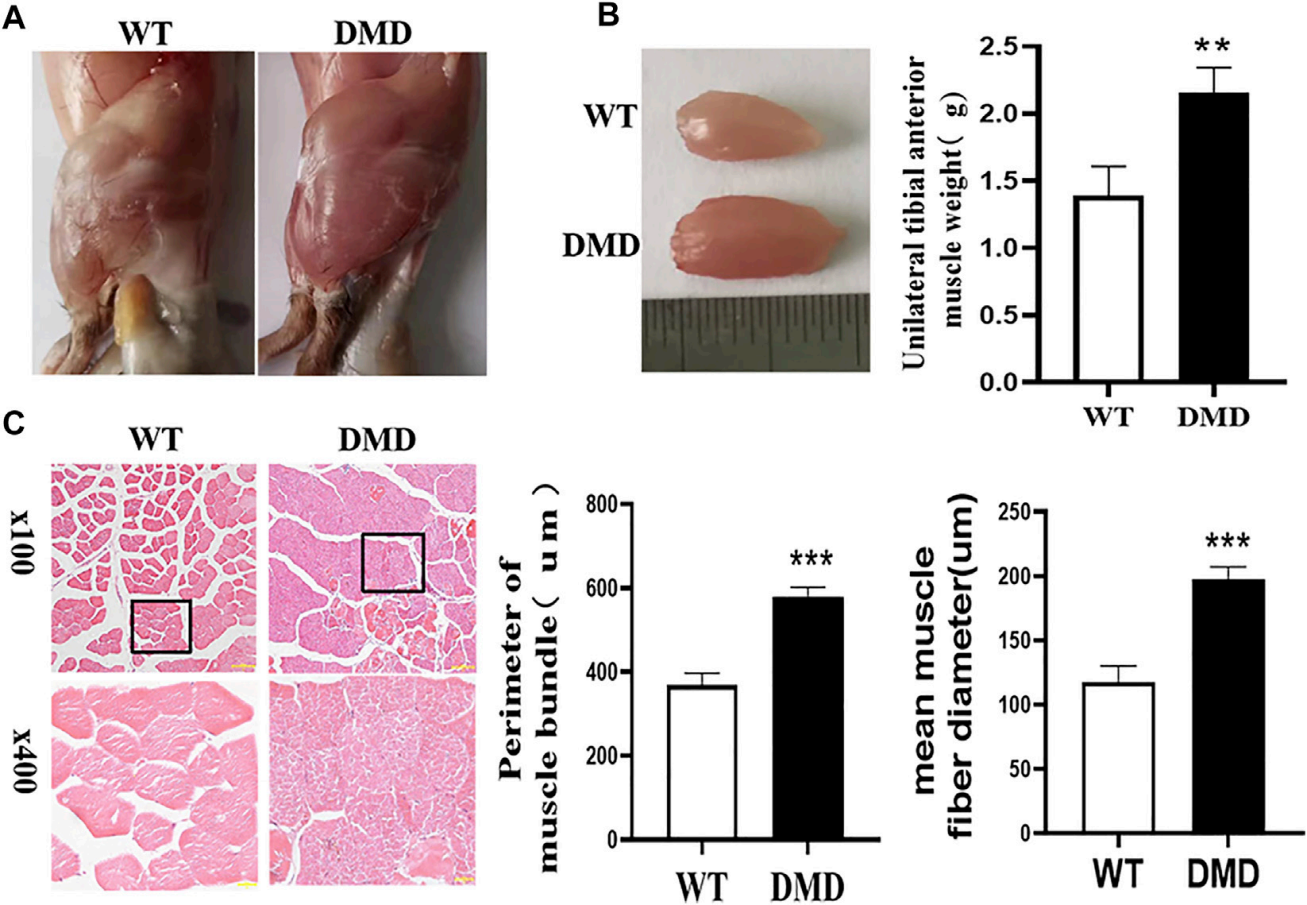
Hypertrophy of tibialis anterior in DMD Mice. **(A)** Overview of lower limb muscles in DMD mice and WT mice. **(B)** The isolated tibialis anterior of WT and DMD, the average weight of unilateral isolated tibialis anterior was measured in DMD and control mice. Data were presented at means ± SEM (*n* = 6) with independent sample *t*-test. **p < 0.01. **(C)** HE staining of bundles on TA. The average circumference of the tibialis anterior was measured in 10 different fields. Quantification and comparison in the mean diameter of the ferret for myofibers were performed. Data were presented at means ± SEM (*n* = 6) with independent sample *t*-test. ***p < 0.001.

Under the macroscopic view, DMD mice have significant hypertrophy of lower limb muscles (Figure 1A). We separated the tibialis anterior muscle of WT and DMD mice and weighed them for comparison, the weight of the tibialis anterior (TA) muscle of the left side showed that the muscle weight of DMD mice is significantly higher than that of WT mice (Figure 1B). There is no significant difference in body weight between WT and DMD mice. The circumference of muscle bundles was measured after HE staining. Feret’s Diameter analysis was used to calculate the average diameter of muscle fibers. The results show that the circumference of muscle bundles in DMD mice was significantly increased when compared to control mice. The former had larger mean muscle fiber diameters than the later (Figure 1C).

**FIGURE 2:**
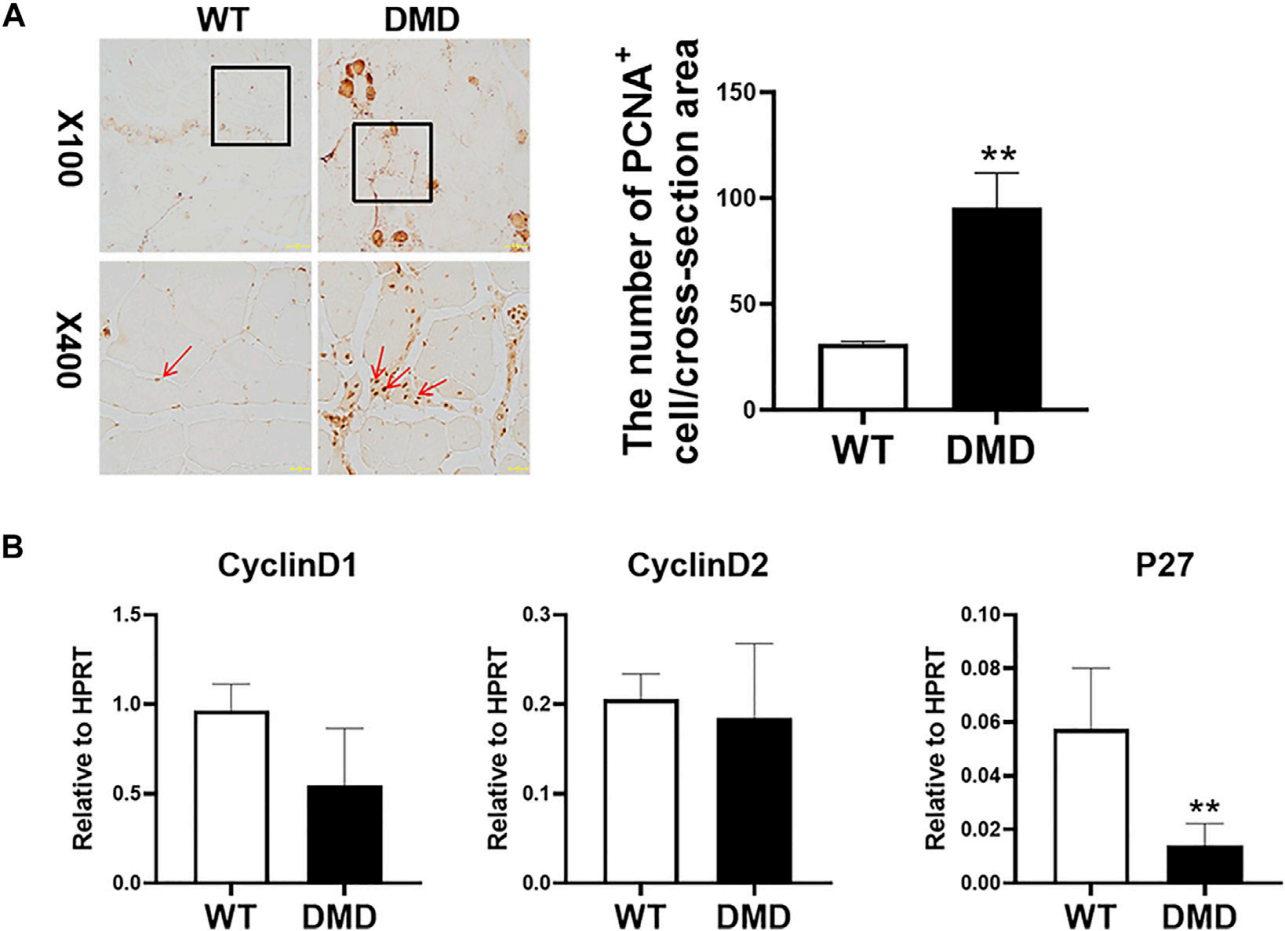
Increase proliferation of muscle cells in tibialis anterior in DMD mice. **(A)** Immunohistochemical staining of mouse muscle cells was performed with the PCNA antibody. PCNA positive cells are shown as brown (red arrow). The PCNA positive cells were counted under microscope. Numbers of PCNA positive cells were demonstrated in the visual field Data were presented at means ± SEM (*n* = 6) with independent sample *t*-test. **p < 0.01. **(B)** Gene expression of tibialis anterior cells in WT and DMD mice was detected by RT-PCR. Data are expressed as the mean ± SEM (*n* = 6). **p < 0.01.

We then measured the proliferation status of muscle cells in tibialis anterior through PCNA immunostaining. As shown in Figure 2A, most of normal muscle cells were in a static state with almost no PCNA positive staining. However, there is a significant increase in PCNA positive staining in DMD mice, which is located between muscle cells and muscle bundles. Statistical analysis of the numbers of these PCNA positive cells suggests that the numbers of positive cells in DMD mice significantly increased when compared to muscles in WT mice. Then, we detected proliferation-related gene expression and found that there was no significant difference in the expression of Cyclin D1 and Cyclin D2 between WT and DMD mice. However, the expression of P27 was significantly decreased in DMD mice when compared to that in WT mice. These data showed that cells from tibialis anterior have increased proliferation in DMD mice compared to that in WT mice (Figure 2B).

### Decreased Numbers of Satellite Cells and Increased Oxygen Free Radical Levels in DMD Mice

**FIGURE 3:**
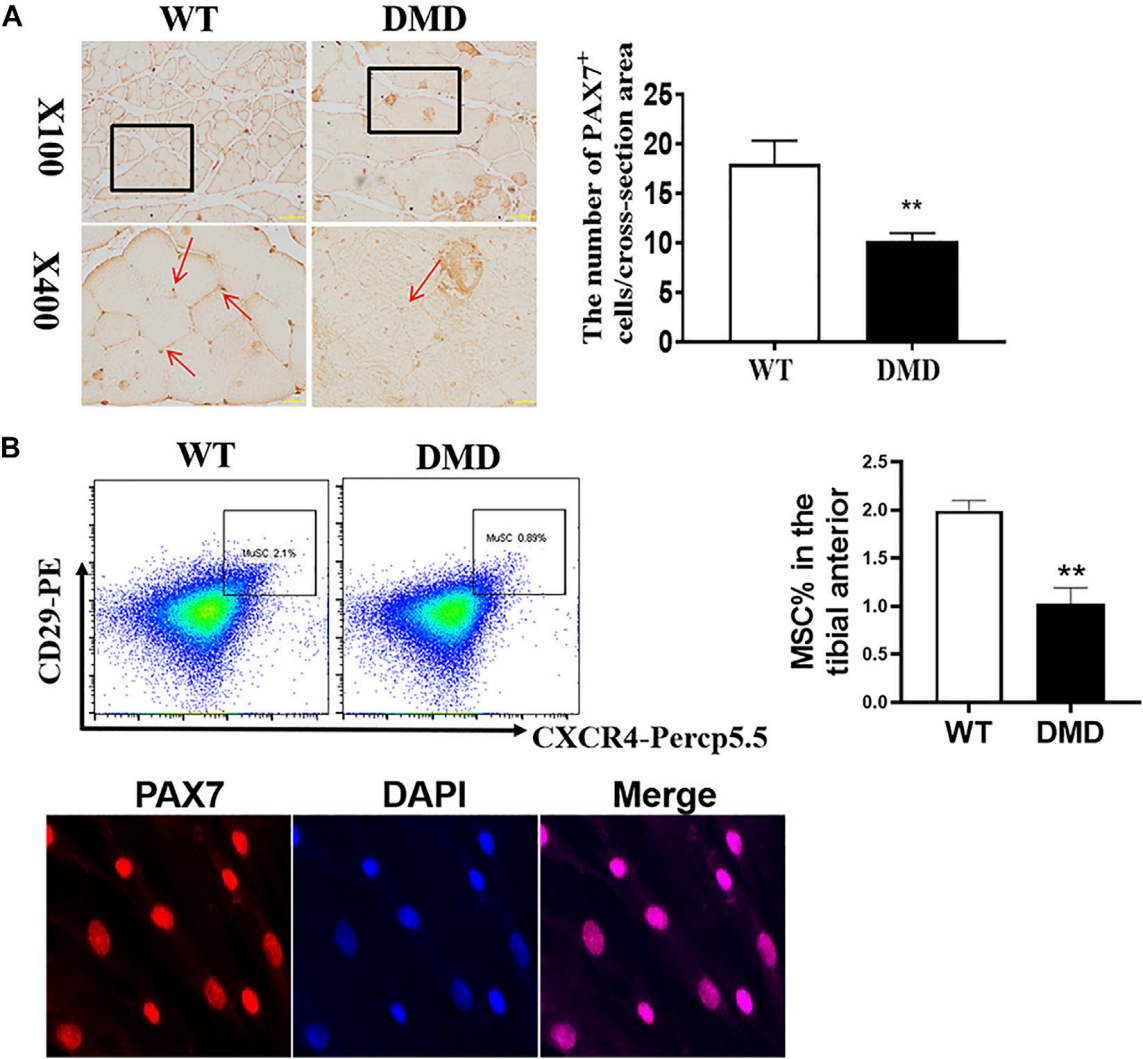
The numbers of muscle stem cells in DMD mice were reduced in the tibial anterior. **(A)** Specific staining of muscle stem cells, PAX7 antibody. The PAX7 positive staining is brown (red arrow, left panel). PAX7 positive cells were counted in each field. The numbers of PAX7 positive cells were demonstrated as means ± SEM (*n* = 6, right panel). **p < 0.01. **(B)** CD11b^−^CD45^−^CD29^+^CXCR4^+^ cells were labeled (flow chart) and sorted out for PAX7 immunostaining. The ratio of WT to DMD muscle stem cells was compared. Data were presented at means ± SEM (*n* = 6) with independent sample *t*-test. **p < 0.01.

In addition to detecting the changes in muscle cells, the numbers of muscle stem cells were also examined in DMD mice. PAX7 was used as a characteristic marker to specific label muscle stem cells. The results showed that the numbers of muscle stem cells in DMD mice were significantly reduced (Figure 3A). Since many factors might be involved in the reduction of muscle stem cells. The increase of oxidative stress is a common factor. Therefore, the separated tibialis anterior bundles were digested into single muscle cells by collagenase. We labeled CD11b^−^CD45^−^CD29^+^CXCR4^+^ cells and separated them by flow cytometry. PAX7 immunofluorescence staining of the sorted cells showed that the sorted cells were stained with PAX7 antibody. We then compared the proportion of labeled cells in tibialis anterior cells of WT and DMD mice, demonstrating that the proportion of muscle stem cells in DMD mice was significantly lower than that in WT mice (Figure 3B).

**FIGURE 4:**
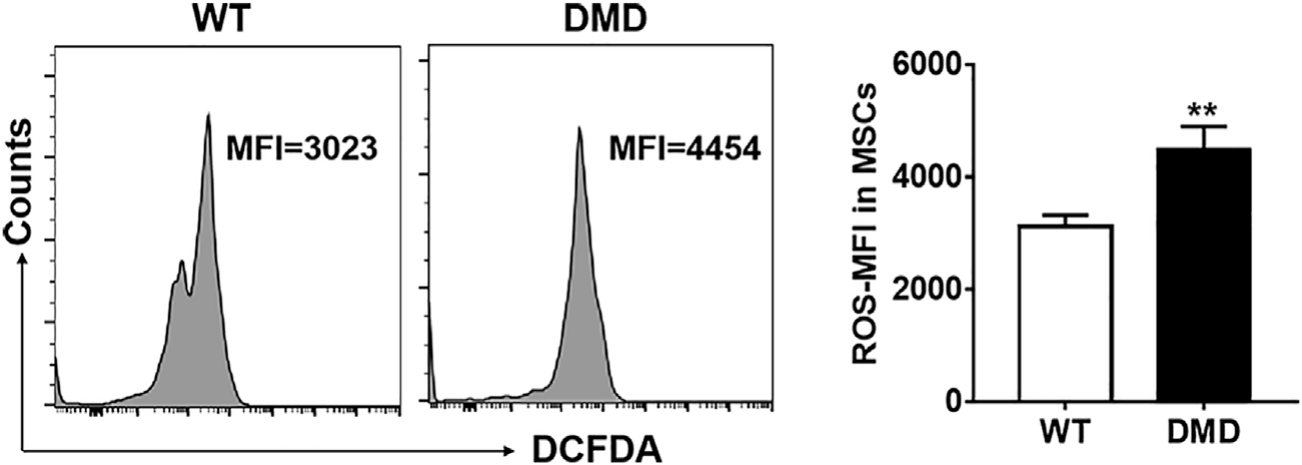
Changes of oxidizing free radicals in muscle stem cells of DMD mice. Free radicals were detected by flow cytometry using the DCFDA probe in muscle stem cells (left panel). The changes of ROS levels in muscle stem cells of WT and DMD mice were compared (*n* = 6, right panel). **p < 0.01.

The DCFDA probe was used to detect the levels of oxygen free radicals in muscle stem cells. As shown in Figure 4, the fluorescence intensity of DCFDA in DMD muscle stem cells significantly increased when compared to that in WT muscle stem cells. These data indicate that elevated levels of oxygen free radicals might be an important factor leading to the damage of muscle stem cells in DMD mice.

### GT3 Treatment Increased the Circumference of Tibialis Anterior and Inhibited Cell Proliferation in DMD Mice

**FIGURE 5:**
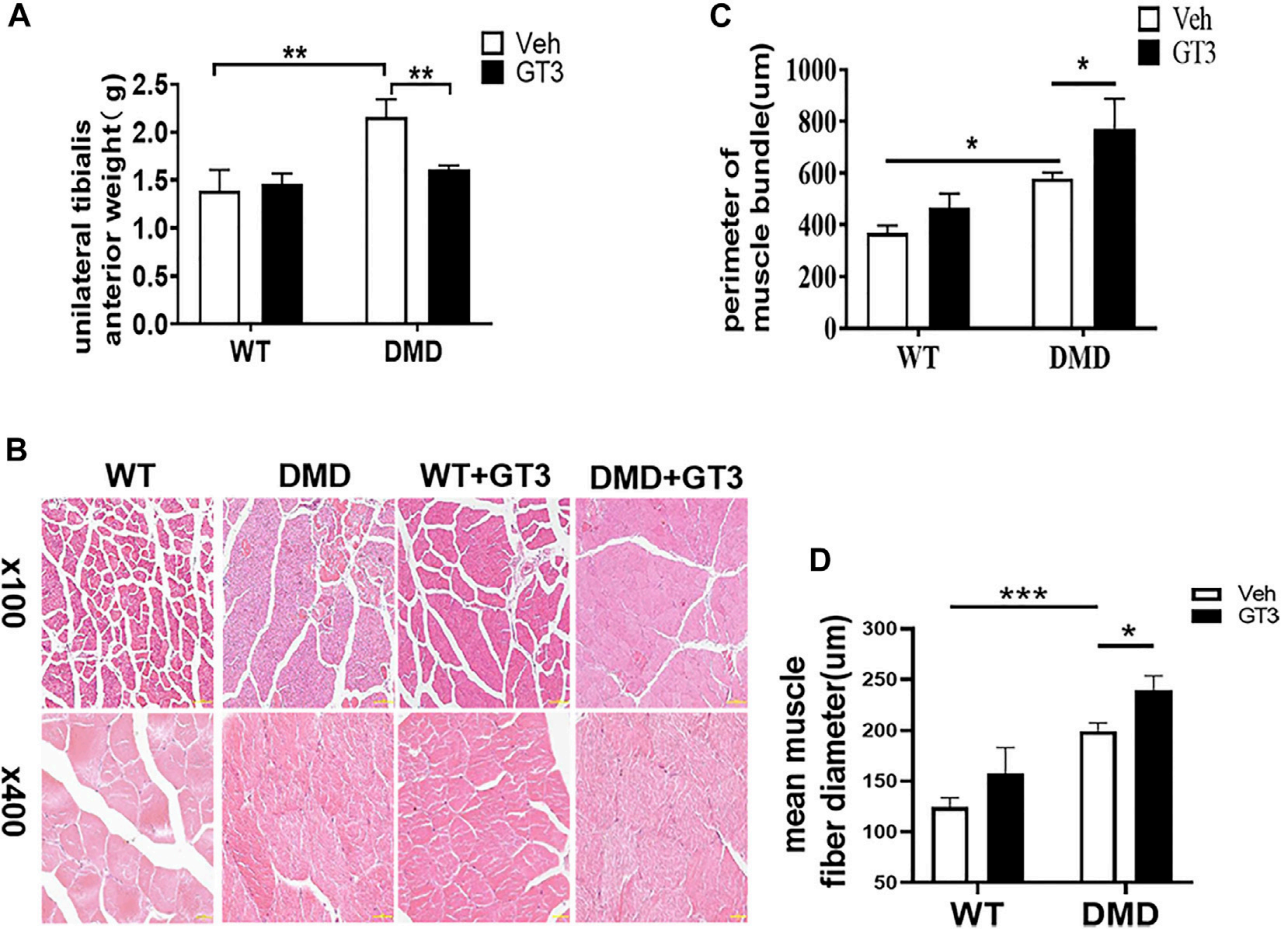
Morphological changes of tibialis anterior in mice treated with GT3. **(A)** The mean wet weight of the left anterior tibial muscle of DMD mice before and after GT3 treatment was compared. Data were presented at means ± SEM (*n* = 6) with two-way ANOVA analysis. **p < 0.01. **(B)** HE staining of tibialis anterior muscle of mice in different treatment groups. **(C)** Comparison of the girth of tibialis anterior muscle in mice of different treatment groups. Data were presented at means ± SEM (*n* = 6) with two-way ANOVA analysis. *p < 0.05. **(D)** Quantification and comparison in the mean diameter of the ferret for myofiber. Data were presented at means ± SEM (*n* = 6) with two-way ANOVA analysis. *p < 0.05, ***p < 0.001.

**FIGURE 6:**
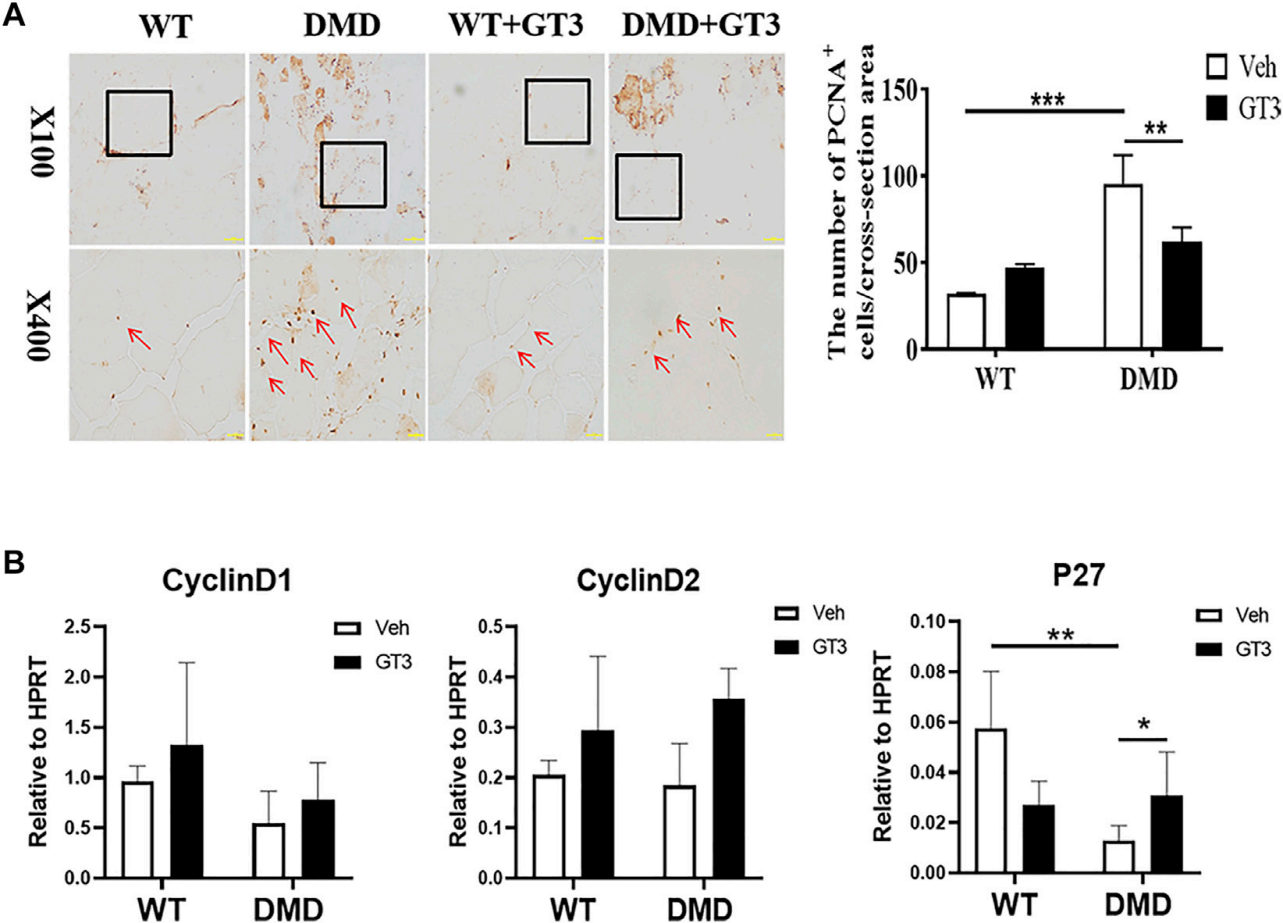
Effects of GT3 on proliferation of muscle cells. **(A)** The tibialis anterior was extracted 48 h after GT3 treatment in 40-week-old WT and DMD mice. PCNA positive cells were stained with brown (red arrow, left panel). Numbers of PCNA positive cells were counted in each field. Data were presented at means ± SEM (*n* = 6) with two-way ANOVA analysis. **p < 0.01, ***p < 0.001. **(B)** Gene expression of tibialis anterior muscle cells was detected by RT-PCR after GT3 treatment. Data were presented at means ± SEM (*n* = 6) with two-way ANOVA analysis. *p < 0.05, **p < 0.01.

It is well known that GT3 has a strong ability to inhibit ROS production. In order to explore the role of GT3 in the injuried muscle of DMD mice, WT and DMD mice were treated with GT3 for 48 h and the tibialis anterior muscle was collected for analysis. GT3 treatment did not affect body weight in WT and DMD mice. However, the weight of the left tibialis anterior in the DMD group after GT3 treatment was significantly reduced compared that in the DMD group after vehicle treatment (Figure 5A). The circumference of muscle bundle was measured, showing that the circumference of the muscle bundle of the TA in DMD mice was increased when compared to the control group after GT3 treatment (Figures 5B,C). In addition, the Feret’s Diameter analysis was also used to compare the mean diameters of tibialis anterior fiber (Figure 5D). After GT3 treatment, the tibialis anterior fibers of DMD mice had larger mean diameters when compared to that of WT mice. Meanwhile, PCNA immunostaining was performed on the muscle. The numbers of PCNA positive cells were counted in each field (Figure 6A). The results showed that the numbers of PCNA positive cells in DMD mice after GT3 treatment were lower than that of the untreated control group, indicating that GT3 treatment can inhibit excessive cell proliferation. Similarly, we compared the gene expression of tibialis anterior cells after GT3 treatment. Our data showed that there was no statistical difference in the expression of Cyclin D1 and Cyclin D2 in tibialis anterior cells after GT3 treatment in WT and DMD mice while the expression of P27 was significantly increased in DMD muscle after GT3 treatment when compared to that in the control group (Figure 6B).

### GT3 Treatment Increased Numbers of Muscle Stem Cells in the Tibialis Anterior of DMD Mice

**FIGURE 7:**
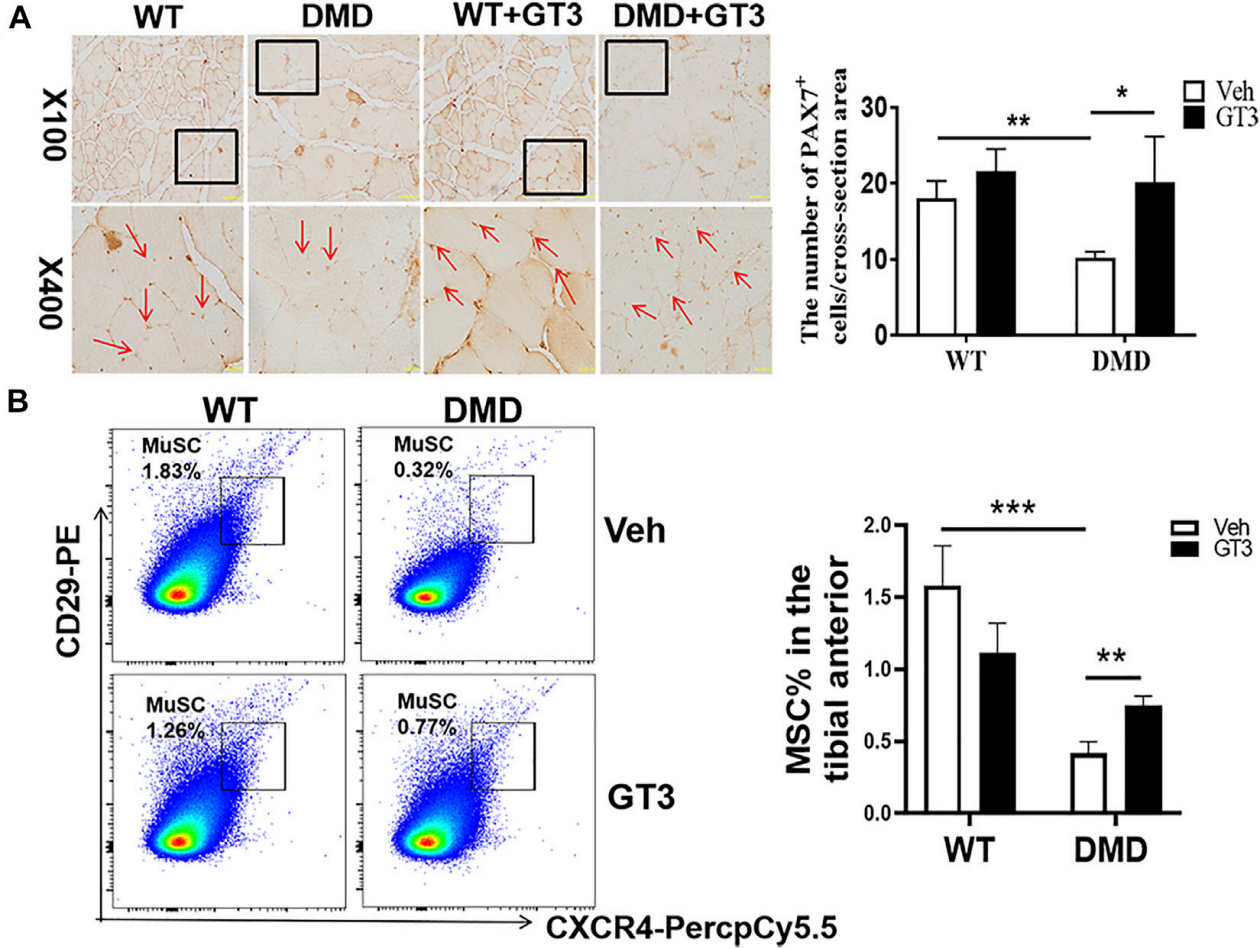
Changes in the numbers of muscle stem cells after GT3 treatment. **(A)** Forty-week-old WT and DMD mice were treated with GT3 for 48 h. The tibialis anterior were taken for PAX7 immunostaining (red arrow). Numbers of PAX7 positive cells were counted. *p < 0.05, **p < 0.01. **(B)** CXCR4 and CD29 flow cytometry antibody were used to label muscle stem cells (left panel). The percentages of CD11b^−^CD45^−^CD29^+^CXCR4^+^ cells were demonstrated in the right panel. **p < 0.01, ***p < 0.001.

PAX7 was used to marker muscle stem cells in the tibialis anterior muscle. As shown in Figure 7A, the numbers of muscle stem cells in DMD mice after GT3 treatment were significantly increased compared with the numbers of untreated control group. After GT3 treatment, the percentages of muscle stem cells in DMD mice were increased, which is consistent with the data from PCNA immunostaining. Collectively, these findings suggest GT3 can increase the numbers of muscle stem cells in DMD mice. In addition, we also demonstrated the same results by labeling muscle stem cells with flow cytometry, showing that GT3 treatment significantly increased percentages of muscle stem cells (Figure7B).

### GT3 Treatment can Improve the Differentiation of Muscle Stem Cells in DMD Mice

**FIGURE 8:**
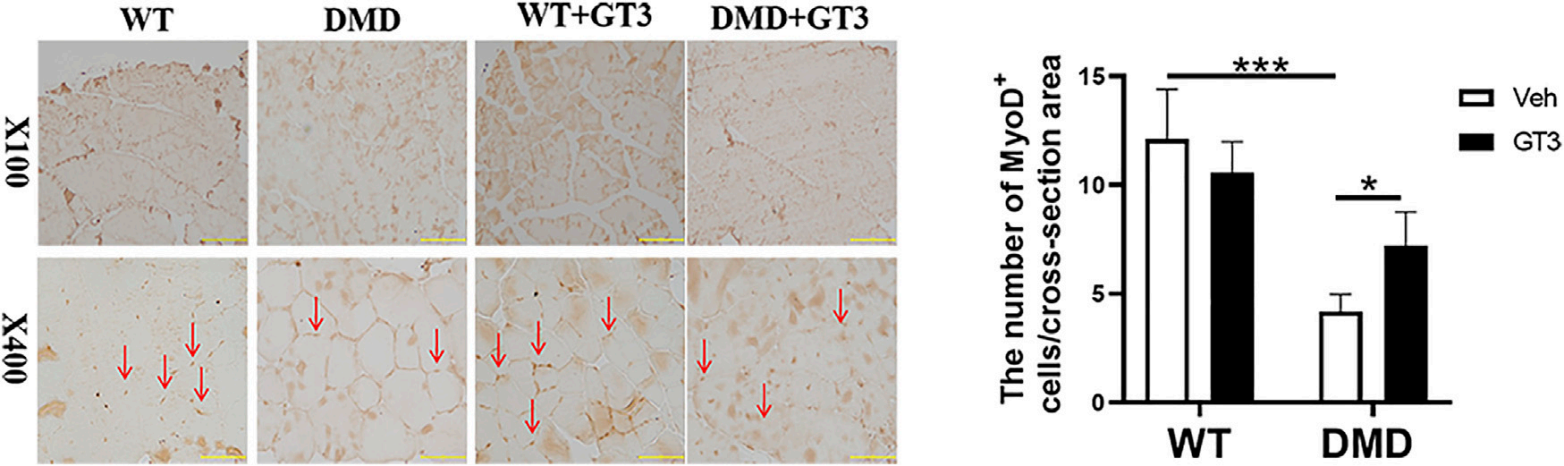
Effects of GT3 on differentiation of muscle stem cells in DMD mice. The tibialis anterior was extracted 48 h after GT3 treatment in 40-week-old WT and DMD mice. MyoD1 positive cells were stained with brown (red arrow, left panel). Numbers of MyoD1 positive cells were counted in each field. Data were presented at means ± SEM (*n* = 6) with two-way ANOVA analysis. *p < 0.05, ***p < 0.001.

In order to verify the effect of GT3 treatment on the function of muscle stem cells in DMD mice, MyoD1 antibody was used to detect their differentiation ability. As shown in Figure 8, the numbers of MyoD1 positive staining in DMD mouse muscle cells were significantly decreased when compared to that in WT mice. However, it was found that there was a significant increase in the numbers of MyoD1 positive cells in the GT3 treatment group of DMD mice when compared to that in the vehicle group. These results suggest that GT3 treatment can improve the differentiation ability of muscle stem cells in DMD mice.

### GT3 Reduces Inflammation and Fibrosis in the Tibialis Anterior of DMD Mice

**FIGURE 9:**
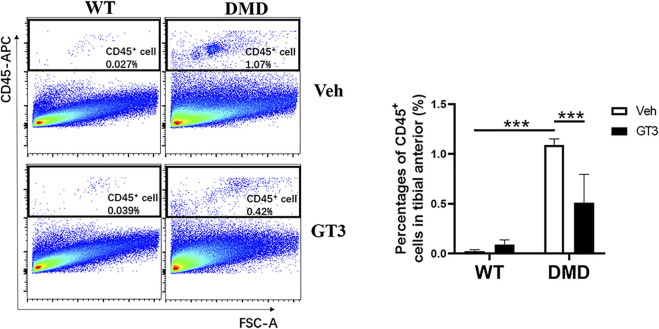
Effect of GT3 on CD45 positive cells in the tibialis anterior of DMD mice. CD45 positive cells were labeled in the tibialis anterior cells and the effect of GT3 treatment on the number of CD45 positive cells was compared. Data were presented at means ± SEM (*n* = 6) with two-way ANOVA analysis. ***p < 0.001.

One of the pathological manifestations of DMD is inflammatory infiltration. CD45 was used as a specific marker of inflammatory response. CD45^+^ cells were analyzed by flow cytometry to measure inflammation levels in the tibialis anterior. As shown in Figure 9, percentages of CD45^+^ cells in DMD mice were significantly increased when compared to those in WT mice. In comparison to the vehicle-treated DMD mice, the percentages of CD45^+^ cells in GT3-treated mice were significantly decreased. These data indicate that GT3 can reduce inflammatory infiltration of tibialis anterior in DMD mice.

**FIGURE 10:**
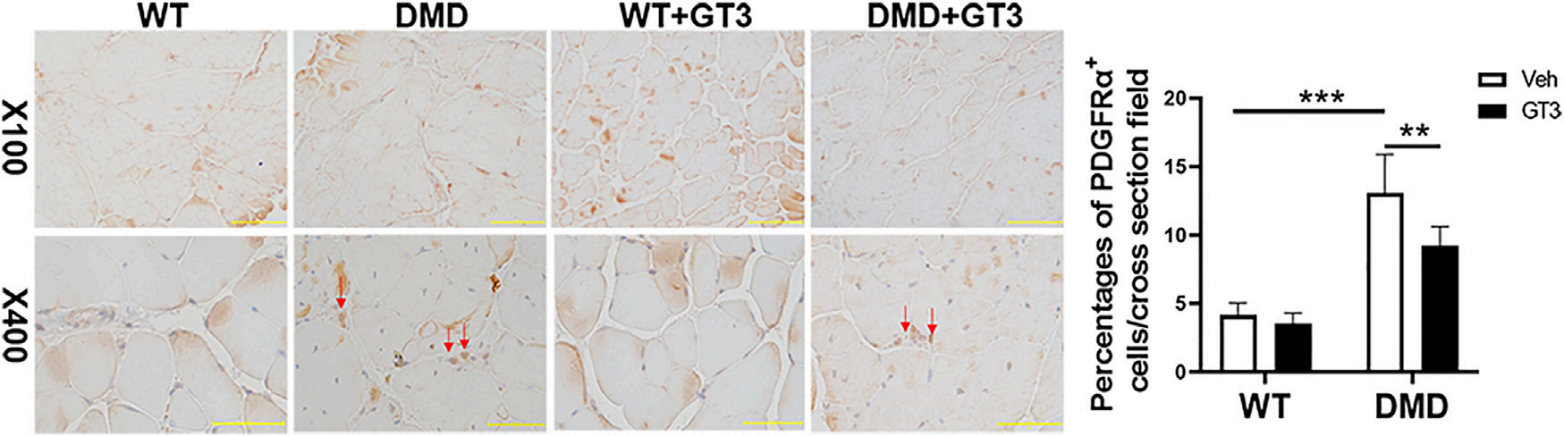
Effect of GT3 treatment on fibrosis of the tibial anterior in DMD mice. DMD mice had more fibro-adipogenic progenitors in the tibialis anterior which compared with WT mice. The percentages of cells were decreased after GT3 treatment in DMD mice. Data were presented at means ± SEM (*n* = 6) with two-way ANOVA analysis. **p < 0.01, ***p < 0.001.

Additionally, increased fibrosis is also a major pathological feature of DMD mice. PDGFRα^+^ cells in skeletal muscle mesenchymal progenitor named fibro-adipogenic progenitors can differentiate into fibroblasts and adipocytes which are thought to play an important role in the fibrosis process (Joe et al., 2010). The data from PDGFRα staining showed that DMD mice had more fibro-adipogenic progenitors in the tibialis anterior when compared to that in WT mice, which was attenuated after GT3 treatment (Figure 10). Taken together, these data display that GT3 treatment inhibits inflammatory infiltration and fibro-adipogenic progenitors in the tibialis anterior caused by loss of dystrophin, which may alleviate the fibrotic symptoms of DMD.

### GT3 Treatment Reduced the Production of Oxidative Free Radicals in Muscle Stem Cells in DMD Mice

**FIGURE 11:**
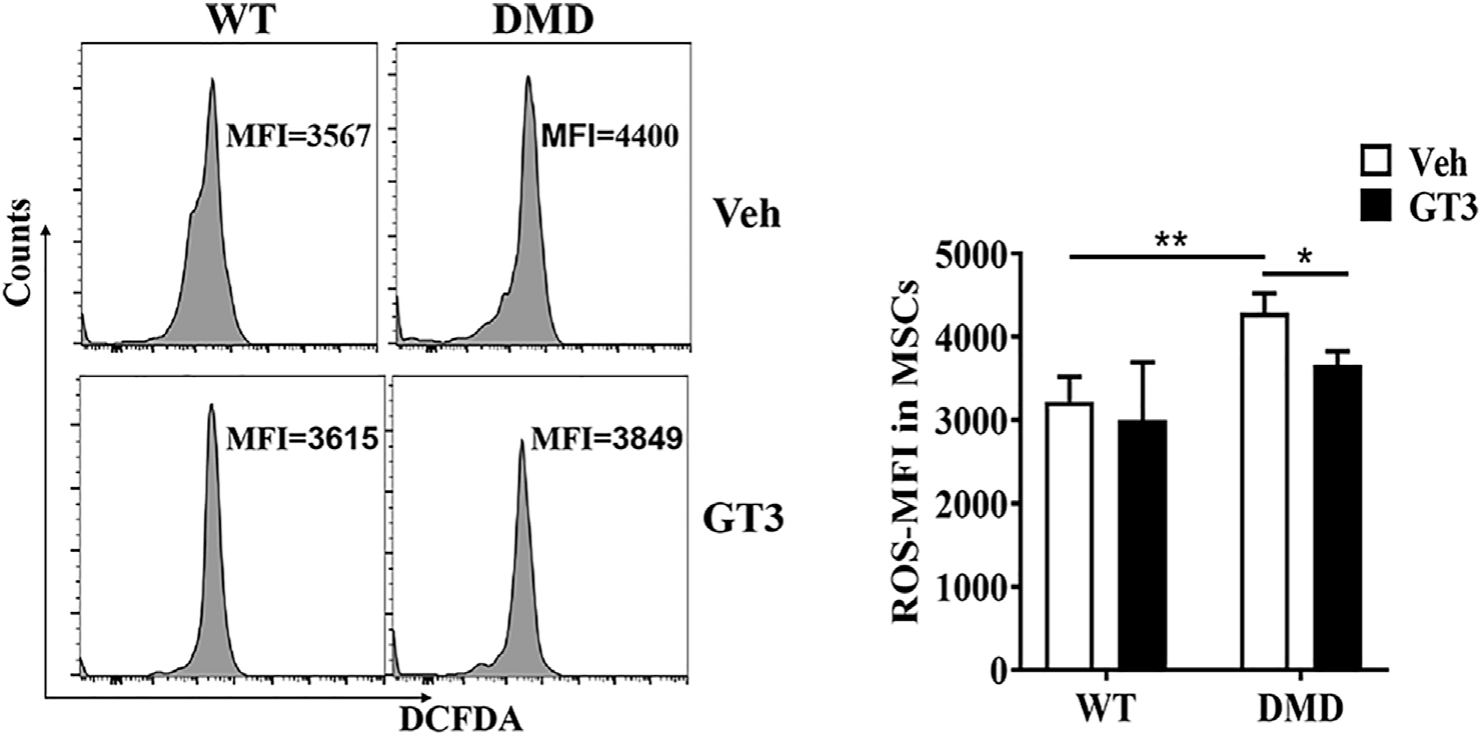
Oxidative free radical levels in DMD mice and WT mice. DCFDA probe was used to detect oxidative free radical in WT and DMD mice by flow cytometry (left panel). Mean fluorescence intensity (MFI) of DCFDA in muscle stem cells were demonstrated. Data were presented at means ± SEM (*n* = 6) with two-way ANOVA analysis. *p < 0.05, **p < 0.01.

We previously demonstrated that muscle stem cells in DMD mice have increased levels of oxygen free radicals. Whether GT3 treatment can decrease oxygen free radicals in DMD mice was then investigated. We used the probe DCFDA to compare the changes of oxidative free radical levels in DMD muscle stem cells before and after GT3 treatment. The results showed that GT3 treatment significantly reduced the levels of oxidized free radicals in muscle stem cells in DMD mice when compared to those in control mice (Figure 11).

## Discussion

The impaired muscle regeneration accompanying the exhaustion of the satellite cell pool is a major feature of DMD. Muscle damage in patients with DMD is manifested by extremely low regenerative capacity, fibrosis, fat deposition, inflammatory infiltration, and fiber atrophy (Bella et al., 2020). At present, there is no effective long-term treatment for DMD. The commonly used clinical method is corticosteroid therapy, but the effect is not satisfactory (Fox et al., 2020). Muscle stem cells are good potential repair factors for damaged muscles due to their omnipotence. Therefore, investigators are exploring the effects of stem cell therapy on DMD. In addition, there are some data showing that DMD progression results in part from a cell-autonomous failure of muscle stem cells to maintain the damage-repair cycle initiated by dystrophin deficiency. It has been shown that maintaining the function of muscle stem cells has therapeutic significance for DMD (Sacco et al., 2010). The responses of muscle stem cells to injury or disease are an activation process. They then re-enter the cell cycle and proliferate to produce a pool of progenitor cells marked by the expression of the myogenic transcription factor MyoD1. The damaged muscle can be replenished and repaired through proliferation and differentiation of muscle stem cells (Liu et al., 2018). We specifically labeled the muscle stem cells in DMD knockout mice and found that numbers of muscle stem cells were significantly reduced. On the contrary, the numbers of proliferating cells were significantly increased when compared to that in normal mice. Previous studies have shown that muscle fibers in DMD mice with less than 52 weeks of age are compensated by vigorous regeneration (Pastoret and Sebille, 1995), which may partly explain the increase in muscle fibers caused by the depletion of muscle stem cells in DMD mice.

In addition, the researchers isolated muscle fibers for cell culture and found that the asymmetric division rate of satellite cells in DMD mice was significantly lower than that in WT mice. These data, suggest that the proportion of cells capable of dividing with self-renewal potential was significantly lower in DMD mice (Wang et al., 2019). Similarly, through isolating muscle stem cells for primary culture to detect their self-renewal and differentiation ability by fluorescence, it was found that muscle stem cells in DMD mice were highly active, leading to excessive consumption. Muscle stem cells in DMD mice have features with high levels of aging cells and ROS production. These abnormalities in DMD mice can be partially ameliorated by administration of nutritional drugs (Zhang et al., 2016). Our present data show that loss of dystrophin results in the increased proliferation of muscle stem cells, which is supported by increasing PCNA^+^ cells and decreasing P27 expression in the tibial anterior of DMD mice. GT3 application *in vivo* can reduce numbers of PCNA^+^ cells and increase P27 expression in DMD mice. The effects and mechanisms of GT3 on the proliferation of muscle stem cells from DMD mice will be further investigated in cell culture condition in our future study.

At present, stem cell therapies have achieved the goal of regenerating damaged tissue by supplementing specific stem cell populations. For patients with DMD, the main therapeutic objective is to reconstruct the muscle stem cell pool containing the myostatin receptors, so as to restore the presence of myostatin expressing muscle fibers and recover muscle function (Shimizu-Motohashi et al., 2019). However, the treatment was not successful in moving from mice to human. Only small amounts of myodystrophy were detected after the transplants (Skuk and Tremblay, 2003). This may be due to immune rejection, limited numbers and migration of injected cells, and a large numbers of cell death after transplantation. These studies demonstrates that there was still a long way to go in trying to improve the development of DMD by allogeneic or autologous stem cell transplantation therapy. Patients with DMD urgently need a new treatment that can directly act on stem cells to repair or alleviate their damage.

The continuous contraction of muscle fibers will cause a significant increase in the production of active oxygen free radicals (Qaisar et al., 2016). These molecules regulate a variety of redox-sensitive signaling pathways and play key roles in cellular processes such as gene expression and protein modification. ROS can modify the proteins on the mitochondrial membrane, leading to osmotic swelling. Excessive ROS can lead to oxidative damage to lipids, protein and DNA, leading to cell death or abnormal cell growth. Oxidative stress and mitochondrial dysfunction play a vital role in the pathophysiological process of muscular dystrophy (Vitiello et al., 2018; Zuo and Pannell, 2015). Membrane fragility caused by muscular dystrophy leads to intracellular Ca^2+^ imbalance, which in turn leads to mitochondrial dysfunction and interleukin-6 (IL-6)-mediated ROS release. The increase in ROS levels in turn induces mitochondrial DNA (mtDNA) damage, resulting in production of large amounts of active oxygen. This vicious circle exacerbates muscle fiber damage and cell death (Verhaart and Aartsma-Rus, 2019). When excessive ROS is produced, it leads to oxidative stress. Reactive oxygen species produced by skeletal muscle contraction will affect muscle adaptation and function. Our analysis of oxidative free radicals in muscle stem cells of WT and DMD mice found that there was a significant increase in ROS production in muscle stem cells of DMD mice. The presence of excessive ROS will lead to a series of pathophysiological changes. ROS overproduction plays an important role in muscle stem cells in the functional damage of DMD mice. Inhibiting ROS production may be an effective measure to improve the function of muscle stem cells in DMD mice.

Phenolic compounds have powerful antioxidant activity, which helps to regulate the peroxidation reaction in the body and controls the production of free radicals. These compounds also help to protect cells from oxidative damage caused by free radicals, which could be generated by risk factors such as radiation exposure (Singh et al., 2014). Both tocopherols and tocotrienols have been shown to be relatively non-toxic, even at higher doses. It has been shown that tocopherols provide significant survival advantages (Suman et al., 2013). Compared with tocopherols, tocotrienols can penetrate cell membranes, removing chain-spreading oxygen free radicals, and capturing singlet oxygen and other active substances (Kulkarni et al., 2012; Zhao et al., 2014).

In the present study, tocotrienol (GT3) is applied to evaluate whether it could reduce excessive oxidative free radicals in muscle stem cells of DMD mice. GT3 can selectively inhibit the PI3K/Akt pathway, Ras/Raf/Erk signal transduction, HMG-CoA reductase and transcription factor NF-κB, and participate in the regulation of various physiological activities of the body (Ahsan et al., 2014). Transcriptomics analyses showed that GT3 regulates the expression of genes related to inflammation, protein transport and cellular redox (Tan et al., 2018; Tang et al., 2019). It has the functions of inducing cell apoptosis, inhibiting colony formation, inhibiting cell survival and cell proliferation (Prasad et al., 2016). In the present study, we found that the levels of oxidative free radicals in muscle stem cells of DMD mice were significantly reduced after application of GT3.This may help to reduce the excessive loss of muscle stem cells during muscle regeneration and thus increase the numbers of muscle stem cells. On the other hand, data from previous studies are consistent with our conclusion that GT3 can improve the differentiation ability of muscle stem cells to repair muscle exhaustion in DMD mice (Zhao et al., 2014; Chen et al., 2015). Therefore, out data suggest that application of GT3 can reduce the ROS production of muscle stem cells and improve their physiological activities in a harmless way. It is worth noting that numbers of CD45^+^ cells were increased in muscle cells of DMD mice, which represents an inflammatory infiltration of muscle cells. This inflammatory response can be partially ameliorated by other drug application (Gordon et al., 2013; Ouisse et al., 2019). This is similar to our findings showing that GT3 treatment significantly decreased percentages of CD45^+^ cells in DMD mice, indicating that GT3 ameliorates inflammatory infiltration. In addition, overactivation of the PDGFRα pathway in DMD mice has previously been shown to lead directly to fibrosis and impede muscle repair (Ieronimakis et al., 2016). These mesenchymal progenitors, expressing PDGFRα in the skeletal muscle, were shown to express pro-fibrotic genes in response to TGF-β (Contreras et al., 2020). These data implicate that PDGFRα^+^ fibro-adipogenic progenitors play an important role in the fibrogenic process (Ieronimakis et al., 2013). Further data displayed that PDGFRα^+^ cells were appeared in the DMD connective tissue thickening region, which partly explains the significance of PDGFRα for muscle fibrosis (Ieronimakis et al., 2016; Contreras et al., 2016). Studies have found that drug treatment can limit the differentiation of mesenchymal progenitors, thereby reducing the transition to PDGFRα^+^ fibro-adipogenic progenitors, controlling fibrosis progression (Zhao et al., 2020). Consistently, the use of GT3 in the present study may partially inhibit the increase of fibro-adipogenic progenitors caused by excessive activation of this pathway to control further development of fibrosis. These data from our study indicatethat GT3 treatment can alleviate the pathological symptoms of DMD.

Our study proves for the first time that the application of GT3 can reduce ROS production of muscle stem cells in DMD mice, which may be beneficial to promote the recovery of muscle stem cell function and muscle morphology in DMD mice. However, it is unknown whether GT3 treatment has long-term effects on muscle stem cells in DMD mice, which will be further investigated in near future. The exact reason why DMD mice did not reproduce the human DMD phenotype is unclear. Some researchers believe that the phenotype in DMD gene knockout mice might be compensated through muscular nutrition by up-regulating a protein similar in structure to dystrophin, which is utrophin (Lu et al., 2014). To overcome this barrier in DMD mice, a new type of double-knockout mouse was created, which knocked out both dystrophin and utrophin genes, making its symptom phenotype more severe and thus clinically close to the phenotype of patients with DMD. In addition, DBA/2J-congenic DMD mdx mice (D2-mdx) have been found to have earlier onset and more pronounced dystrophy phenotype which is closer to clinicopathological characterization (Coley et al., 2016; Hammers et al., 2020). Therefore, D2-mdx is increasingly used in the research of DMD instead of original model of C57/BL phenotype.

## Data Availability

The original contributions presented in the study are included in the article/Supplementary Material, further inquiries can be directed to the corresponding author.
