# Supplementary material for: The Protective Effects of γ-Tocotrienol on Muscle Stem Cells Through Inhibiting Reactive Oxidative Stress Production
Source: Front Cell Dev Biol. 2022 Mar 15;10:820520. doi: 10.3389/fcell.2022.820520 (PMC8965065; doi:10.3389/fcell.2022.820520)
Supplement: Supplementary file 1 [file DataSheet2.PDF]

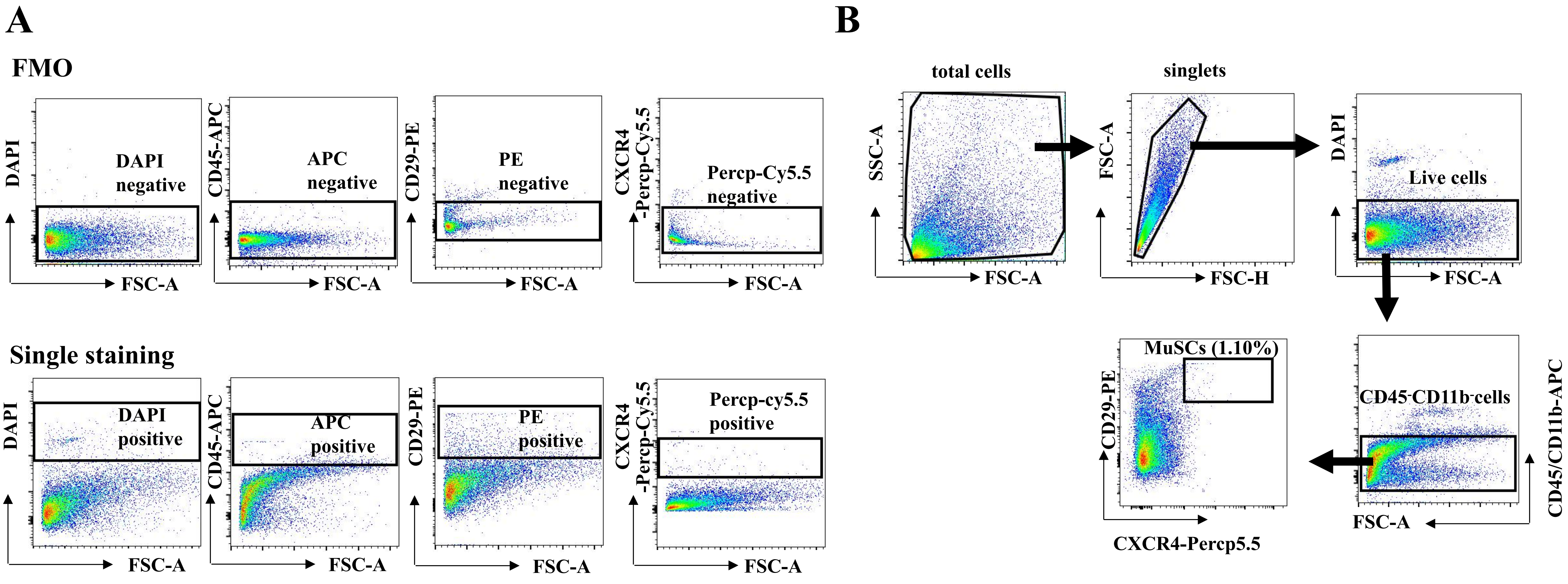

Figure S1 Representative gating strategy of muscle stem cells by flow cytometry

A. Fluorescence minus one (FMO) controls and single staining for the indicated antibodies were used to setup negative and positive gates for different colors. Dead cells were excluded by DAPI staining.

B. Muscle stem cells (MuSCs) gating strategy. FSC-A and FSC-H were used to doublet discrimination to gate singlets. DAPI was used to exclude dead cells. $DAPI^{-}CD45^{-}CD11b^{-}CD29^{+}CXCR4^{+}$ cells were defined as MuSCs.
