# Supplementary material for: The Protective Effects of γ-Tocotrienol on Muscle Stem Cells Through Inhibiting Reactive Oxidative Stress Production
Source: Front Cell Dev Biol. 2022 Mar 15;10:820520. doi: 10.3389/fcell.2022.820520 (PMC8965065; doi:10.3389/fcell.2022.820520)
Supplement: Supplementary file 2 [file DataSheet1.PDF]

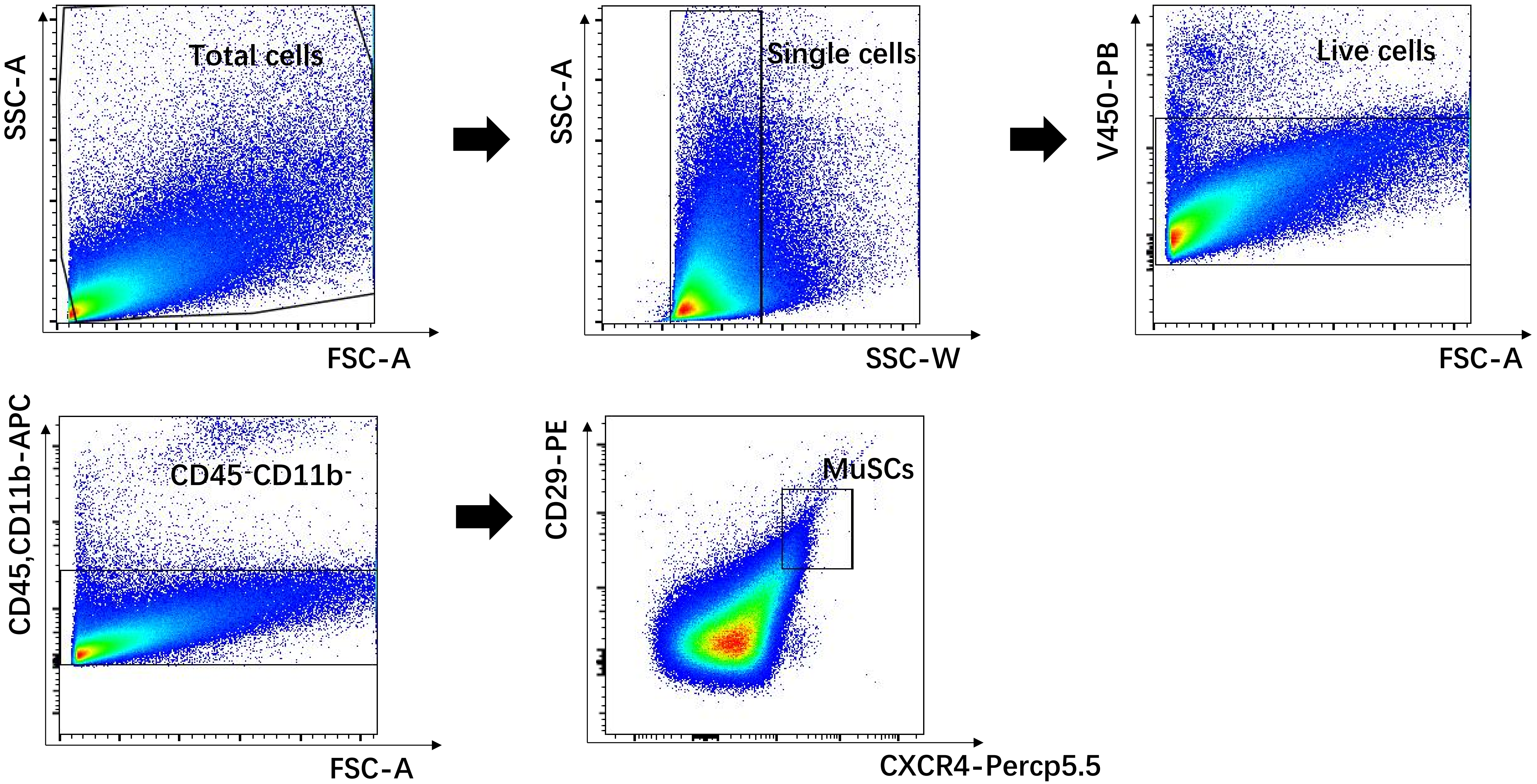

Figure S1 Flow staining gating strategy for analyzing muscle stem cells.

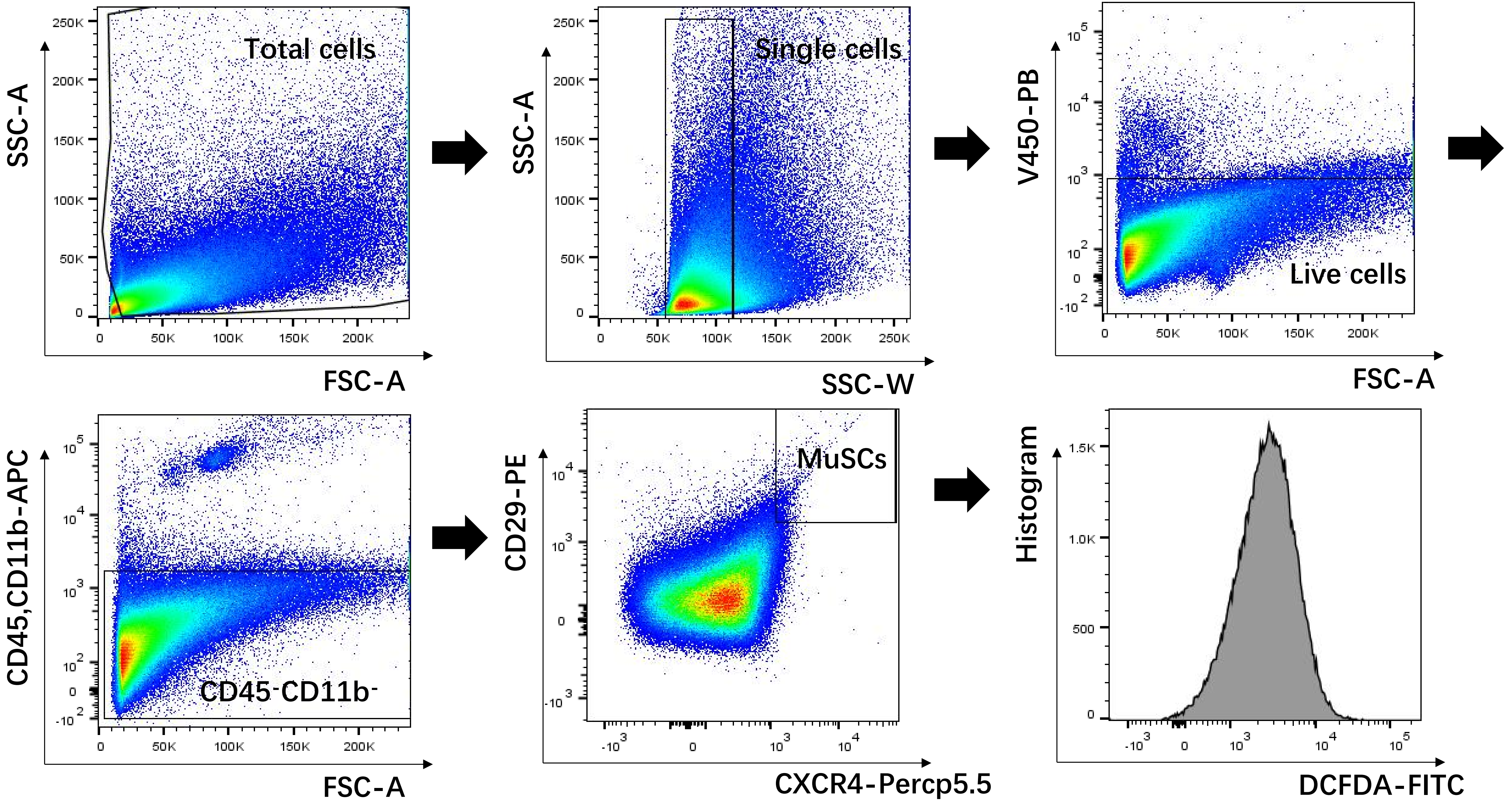

Figure S2 Flow staining gating strategy for analyzing ROS production using the DCFDA probe.

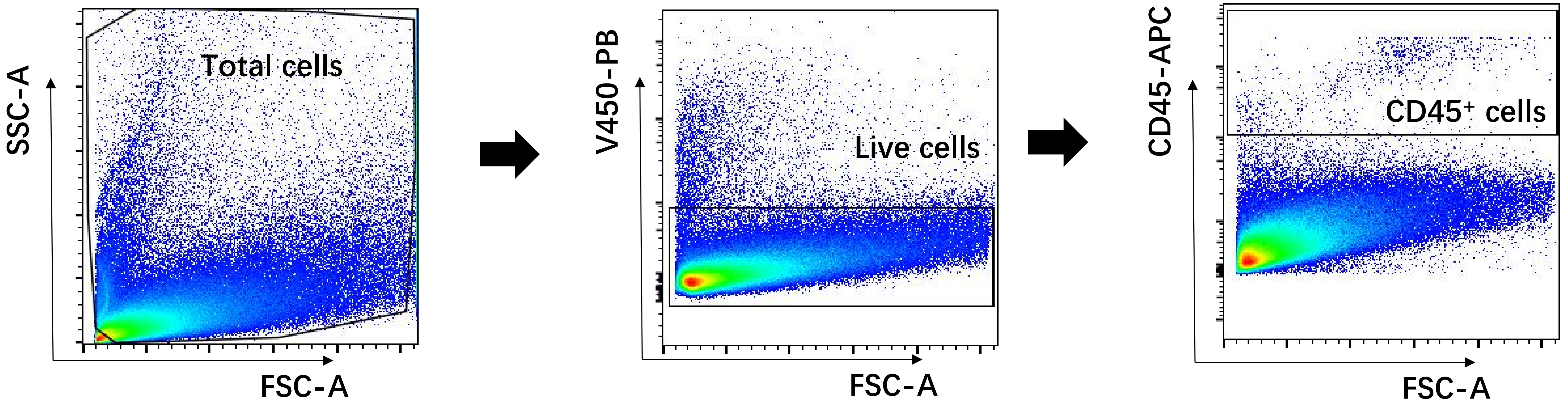

Figure S3 Flow staining gating strategy for analyzing CD45 positive cells
